# Supplementary material for: Longitudinal plasma proteome profiling reveals the diversity of biomarkers for diagnosis and cetuximab therapy response of colorectal cancer
Source: Nat Commun. 2024 Feb 1;15:980. doi: 10.1038/s41467-024-44911-1 (PMC10834432; doi:10.1038/s41467-024-44911-1)
Supplement: Supplementary file 1 — Supplementary Information [file 41467_2024_44911_MOESM1_ESM.pdf]

Supplementary Information

Longitudinal plasma proteome profiling reveals the diversity of biomarkers for diagnosis and cetuximab therapy response of colorectal cancer

Yan Li[1,#], Bing Wang[1,#], Wentao Yang[2,3,#], Fahan Ma[1,#], Jianling Zou[2,3,#], Kai Li[1], Subei Tan[1], Jinwen Feng[1], Yunzhi Wang[1], Zhaoyu Qin[1], Zhiyu Chen[2,3,*] and Chen Ding[1,*]

[1]State Key Laboratory of Genetic Engineering and Collaborative Innovation Center for Genetics and Development, School of Life Sciences, Institutes of Biomedical Sciences, Human Phenome Institute, Zhongshan Hospital, Fudan University, Shanghai, China

[2]Department of Gastrointestinal Medical Oncology, Fudan University Shanghai Cancer Center, Shanghai, China

[3]Department of Oncology, Shanghai Medical College, Fudan University, Shanghai, China

[#]These authors contributed equally

[*]Correspondence: chanhj75@aliyun.com (Z.C.); chend@fudan.edu.cn (C.D.)

Supplementary Figure 1

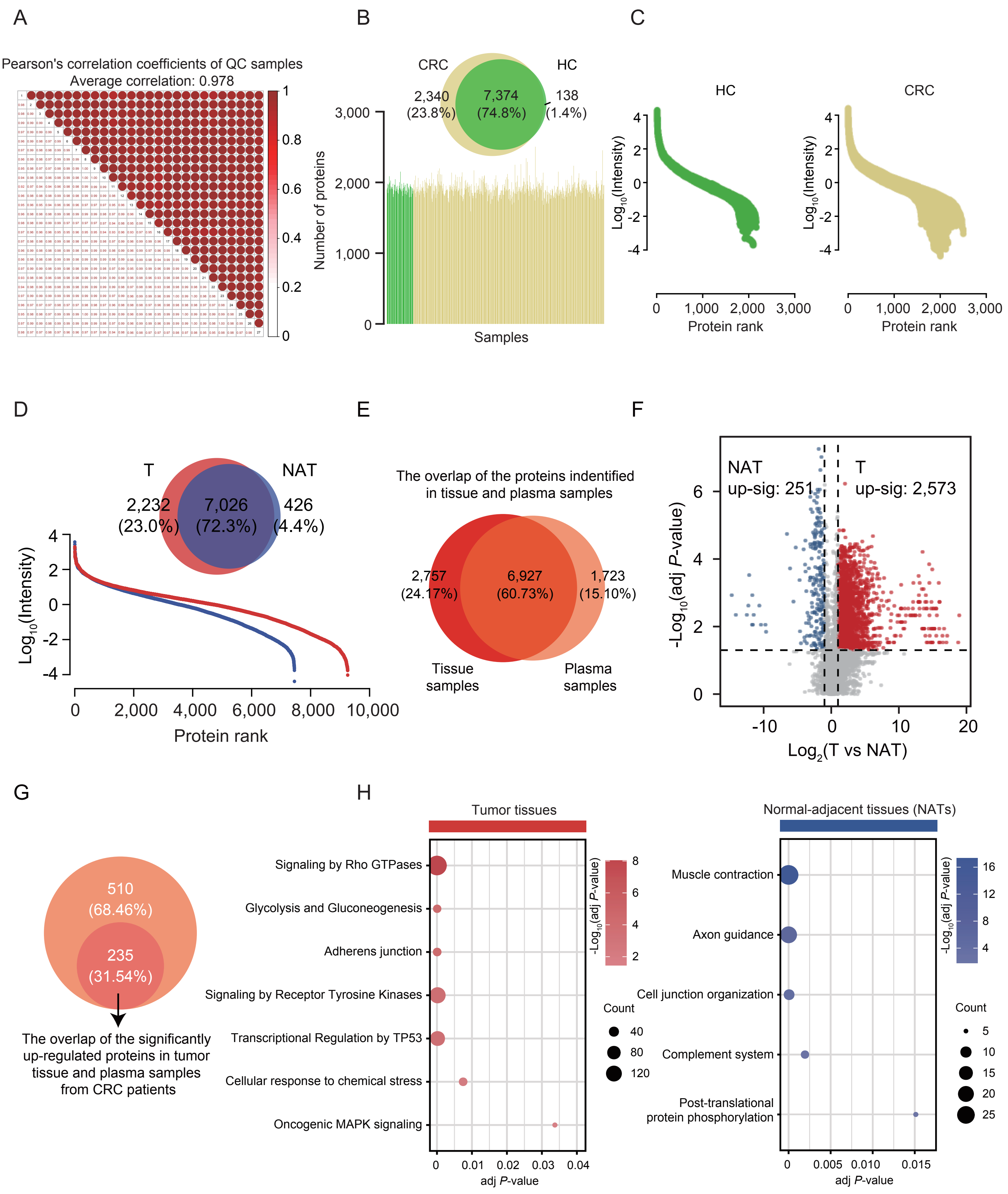

Supplementary Figure 2

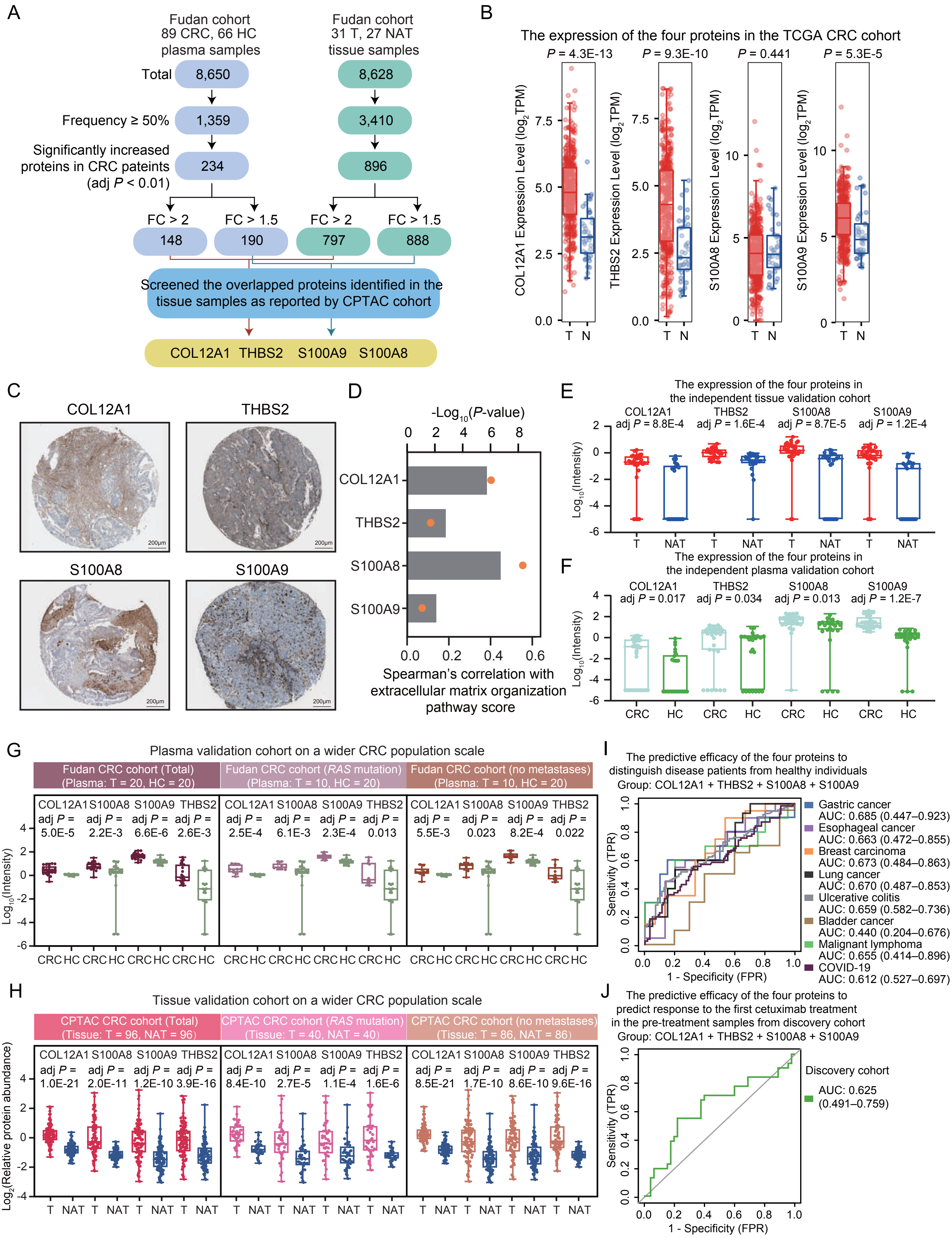

Supplementary Figure 3

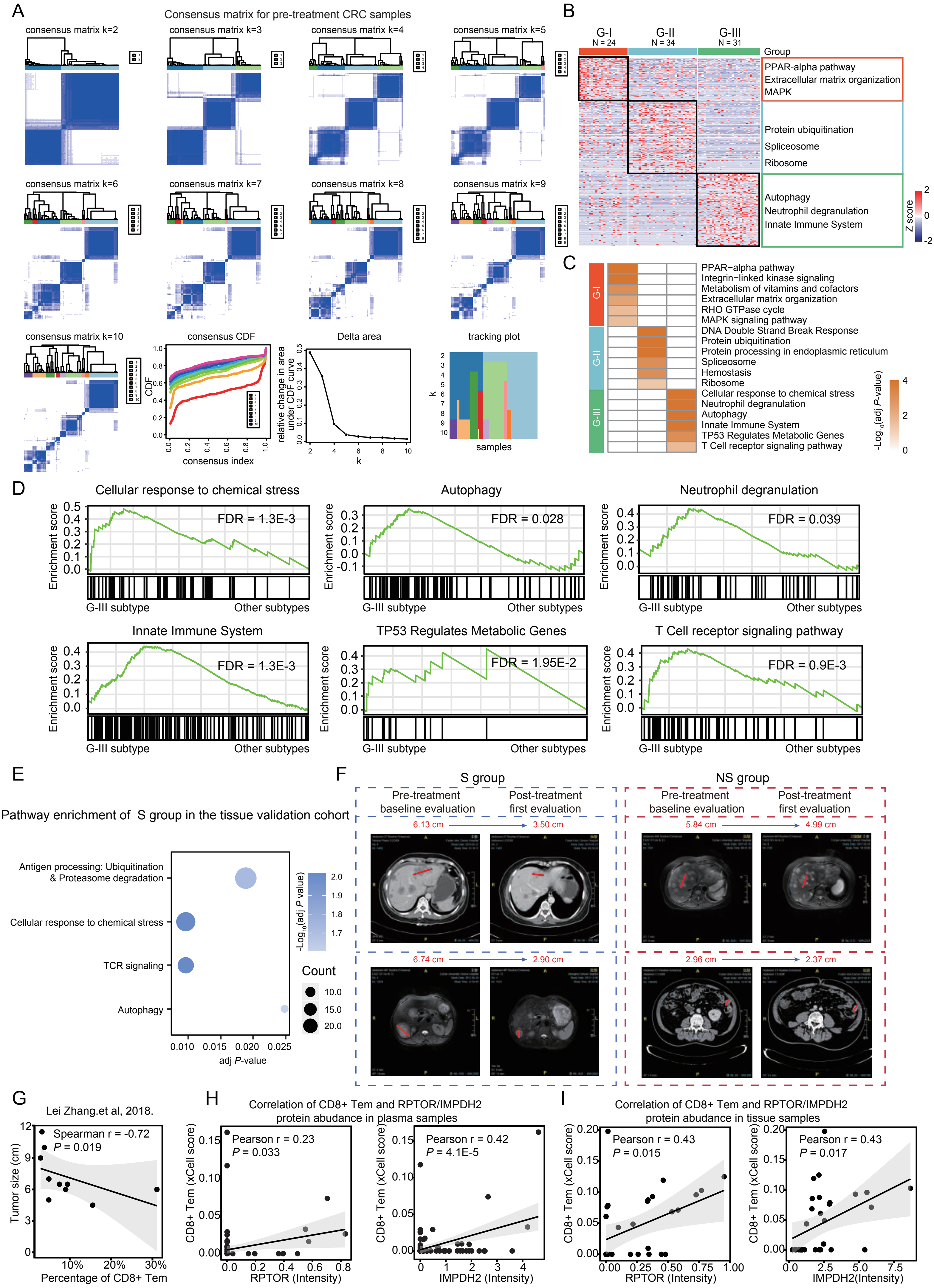

Supplementary Figure 4

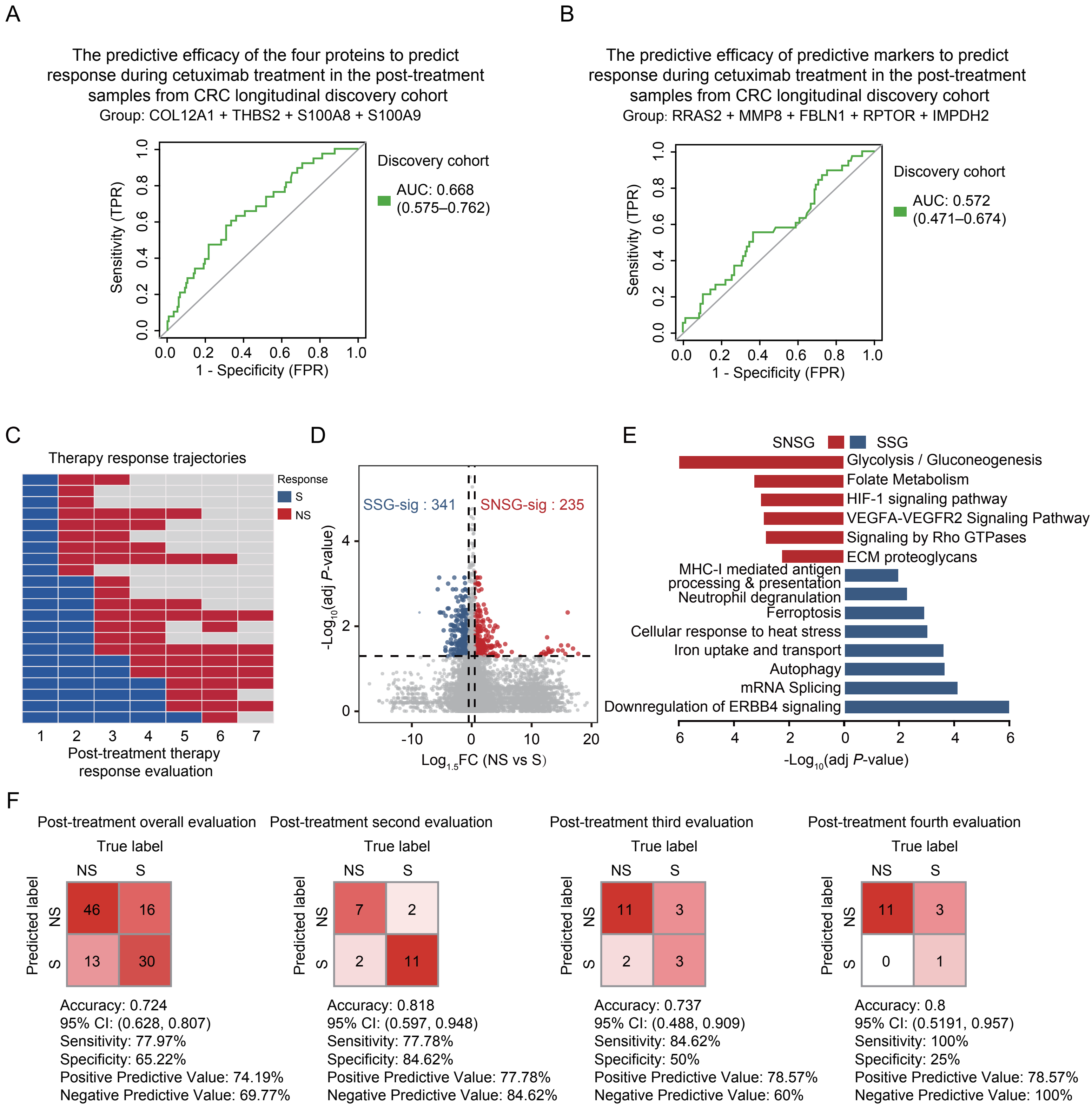

Supplementary Figure 5

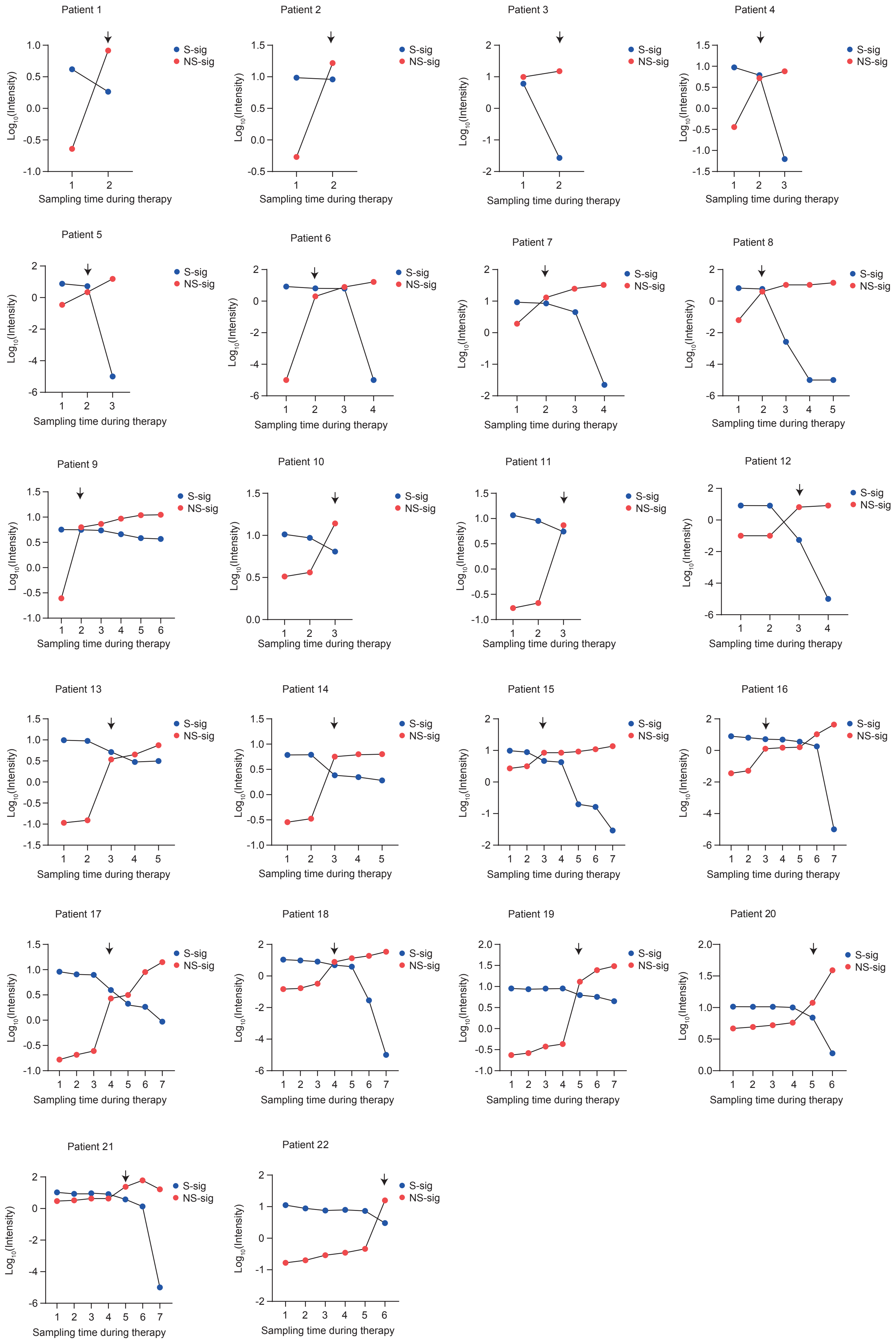

Supplementary Figure 6

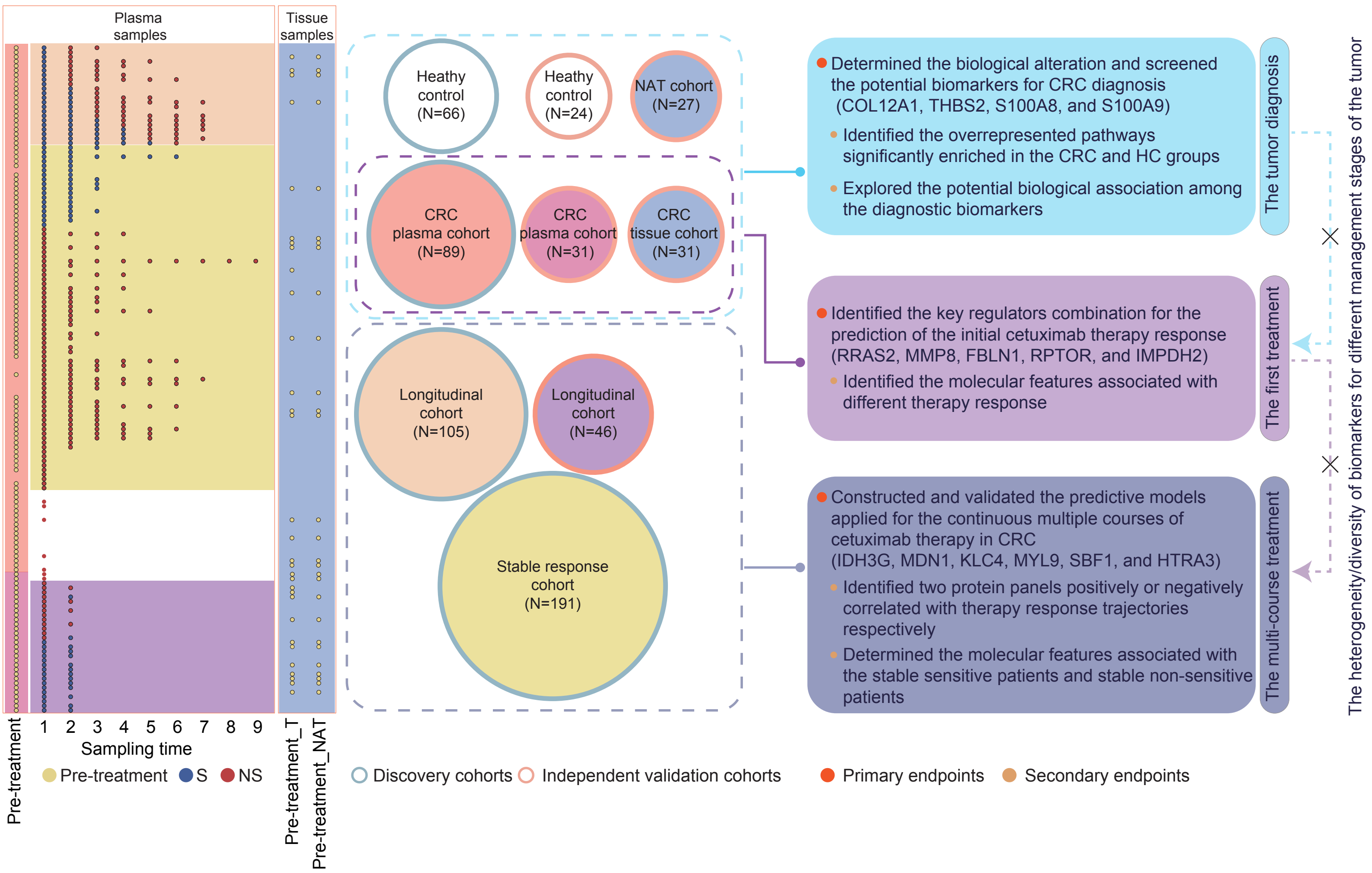

Supplementary Figure Legends

Supplementary Fig. 1. Proteomic identification and quality control in the mass spectrometry platform.

(A) Longitudinal quality control of MS using tryptic digests of the mixture of all plasma samples. The bottom-left half of the panel represents the pairwise Pearson's correlation coefficients of the samples (two-sided Pearson's correlation test), and the top-right half of the panel depicts the pairwise scatter plots from the same comparison.

(B) Venn diagram showing the protein overlap of CRC and HC. Barplots for the identified protein numbers in healthy control (N = 66) and CRC cohort (N = 474).

(C) The dynamic range of protein identification of each sample was shown according to the descending sort of protein abundance of each sample grouped into HC and CRC groups.

(D) Venn diagram showing the protein overlap of tumors and NATs. The dynamic range of protein identification of Tumors and NATs was shown according to the descending sort of protein abundance of each sample grouped into T and NAT groups.

(E) The overlap of the proteins identified in tissue and plasma samples.

(F) Volcano showing the differential expressed proteins between T and NAT groups (two-sided Wilcoxon rank-sum test). Red, upregulated proteins in T group; blue, upregulated proteins in NAT group.

(G) The overlap of the significantly up-regulated proteins in tumor tissues and plasma samples from CRC patients.

(H) Bubble plots showing the CPDB pathway enrichment (two-sided Fisher's exact test) of tumors and NATs.

Supplementary Fig. 2. The differential expression of the four proteins.

(A) The screening flow of proteins with 2-fold and 1.5-fold differential expression.

(B and C) The expression of the four proteins (COL12A1, THBS2, S100A8, and S100A9) are validated by the TCGA CRC cohort (B) and HPA IHC Staining Data (C). The scale bar indicates 200 μm.

(D) Correlation analysis of ssGSEA pathway scores of extracellular matrix organization with the

four proteins (two-sided Spearman's correlation test).

(E and F) The boxplot showing the differential expression of the four proteins between the T and NAT groups in the tissue validation cohort (E), and between the CRC and HC groups in the plasma validation cohort (F) (two-sided Wilcoxon rank-sum test, adj *P*-value < 0.05). Boxplots show median (central line), upper and lower quartiles (box limits), 1.5×interquartile range (whiskers).

(G and H) The boxplots showing the up-regulation of the four proteins in the total CRC patients, the CRC patients with *RAS* mutation, the CRC patients with no metastases in the Fudan plasma validation cohort (G) and the CPTAC tissue validation cohort (H) on a wider CRC population scale (two-sided Wilcoxon rank-sum test, adj *P*-value < 0.05). Boxplots show median (central line), upper and lower quartiles (box limits), 1.5×interquartile range (whiskers).

(I) ROC curves of the four proteins to distinguish patients with different cancers or diseases from healthy controls in the multi-cancer plasma independent cohort.

(J) ROC curves of diagnostic biomarkers to distinguish sensitive patients from non-sensitive patients in the first cetuximab treatment.

Supplementary Fig. 3. Consensus clustering analysis of CRC cohort identified three proteomic subtypes.

(A) The consensus clustering analysis of 89 therapy-naive CRC samples and three subtypes were generated. k was tested from 2 to 10. Consensus matrices, as well as the consensus cumulative distribution function (CDF) plot, delta area (change in CDF area) plot, and tracking plot are shown.

(B) The heatmap depicts the relative abundance (Z score) of the signature proteins in three subtypes of CRC pre-treatment samples. N (G-I) = 24, N (G-II) = 34, and N (G-III) = 31. Biological functions related to these signature proteins are denoted on the right.

(C) The CPDB pathway enrichment (two-sided Fisher's exact test) in the three proteomic subtypes (G-I, G-II, and G-III).

(D) GSEA showing the enrichment of cellular response to chemical stress, autophagy, neutrophil degranulation, innate immune system, TP53 regulates metabolic genes, and T cell receptor signaling pathways in G-III subtype compared with the other two subtypes. FDR < 0.05

is considered statistically significant.

(E) The computed tomography (CT) or magnetic resonance imaging (MRI) scanning features of the representative patients in S and NS group.

(F) Correlation analysis of the proportion of CD8+Tem cell and tumor size in the single-cell transcriptome data (two-sided Pearson’s correlation test). $P < 0.05$ is considered statistically significant.

(G and H) Correlation of CD8+Tem and RPTOR / IMPDH2 protein abundance in the plasma samples (G) and tissue samples (H) (two-sided Pearson’s correlation test). $P < 0.05$ is considered statistically significant.

Supplementary Fig. 4. The analysis of the predictive models applied for the cetuximab treatment response trajectories among continuous multiple courses in the longitudinal cohort.

(A and B) The prediction effect of the diagnostic biomarkers (A), and the key proteins involved in the potential resistant/sensitive mechanism (B) in the CRC longitudinal discovery cohort.

(C) The CRC patients with sensitive (S) /non-sensitive (NS) during the courses of cetuximab treatment. Red: non-sensitive patients (NS); blue: sensitive patients (S).

(D) Volcano showing differential expression of proteins between stable sensitive group (SSG) and stable non-sensitive group (SNSG) in the CRC longitudinal discovery cohort (two-sided Wilcoxon rank-sum test). The adj *P*-value < 0.05 is considered statistically significant.

(E) The CPDB pathway enrichment (two-sided Fisher’s exact test) in SSG and SNSG in the CRC longitudinal discovery cohort.

(F) The classification error matrix of the predictive model to predict the response of cetuximab treatment in different courses.

Supplementary Fig. 5. The dynamic fluctuation of the biomarkers for the longitudinal response prediction across multiple courses during cetuximab treatment. S-sig: MDN1, KLC4, and IDH3G; NS-sig: SBF1, HTRA3, and MYL9. The arrow indicates the resistance emerged. The x axis represents the sampling time: “1” means the first sampling after receiving one course treatment; “2” means that the second sampling after receiving two course

treatments; “3” is defined as the third sampling after receiving three course treatments; “4” is defined as the fourth sampling after receiving four course treatments; “5” is defined as the fifth sampling after receiving five course treatments; “6” is defined as the sixth sampling after receiving six course treatments; “7” is defined as the seventh sampling after receiving seven course treatments. The y axis represents the protein intensity (log_{10} transformed).

Supplementary Fig. 6. The diagram summarizing the connection of clinical samples, data analysis, and major findings.
