# Supplementary material for: Longitudinal plasma proteome profiling reveals the diversity of biomarkers for diagnosis and cetuximab therapy response of colorectal cancer
Source: Nat Commun. 2024 Feb 1;15:980. doi: 10.1038/s41467-024-44911-1 (PMC10834432; doi:10.1038/s41467-024-44911-1)
Supplement: Supplementary file 3 — Reporting Summary [file 41467_2024_44911_MOESM3_ESM.pdf]

nature portfolio

Corresponding author(s): Chen Ding

Last updated by author(s): Nov 24, 2023

Reporting Summary

Nature Portfolio wishes to improve the reproducibility of the work that we publish. This form provides structure for consistency and transparency in reporting. For further information on Nature Portfolio policies, see our Editorial Policies and the Editorial Policy Checklist.

Statistics

For all statistical analyses, confirm that the following items are present in the figure legend, table legend, main text, or Methods section.

n/a	Confirmed	
☐	☒	The exact sample size (*n*) for each experimental group/condition, given as a discrete number and unit of measurement
☐	☒	A statement on whether measurements were taken from distinct samples or whether the same sample was measured repeatedly
☐	☒	The statistical test(s) used AND whether they are one- or two-sided<br>*Only common tests should be described solely by name; describe more complex techniques in the Methods section.*
☐	☒	A description of all covariates tested
☐	☒	A description of any assumptions or corrections, such as tests of normality and adjustment for multiple comparisons
☐	☒	A full description of the statistical parameters including central tendency (e.g. means) or other basic estimates (e.g. regression coefficient) AND variation (e.g. standard deviation) or associated estimates of uncertainty (e.g. confidence intervals)
☐	☒	For null hypothesis testing, the test statistic (e.g. *F*, *t*, *r*) with confidence intervals, effect sizes, degrees of freedom and *P* value noted<br>*Give P values as exact values whenever suitable.*
☒	☐	For Bayesian analysis, information on the choice of priors and Markov chain Monte Carlo settings
☐	☒	For hierarchical and complex designs, identification of the appropriate level for tests and full reporting of outcomes
☐	☒	Estimates of effect sizes (e.g. Cohen's *d*, Pearson's *r*), indicating how they were calculated

Our web collection on statistics for biologists contains articles on many of the points above.

Software and code

Policy information about availability of computer code

Data collection	Samples were analysed on a Q Exactive HF-X Hybrid Quadrupole-Orbitrap Mass Spectrometer (Thermo Fisher Scientific, Rockford, IL, USA) coupled with a high-performance liquid chromatography system (EASY nLC 1200, Thermo Fisher Scientific). The mass spectrometry data were acquired using the Xcalibur software v2.2 (Thermo Fischer Scientific).
Data analysis	Database searching for the mass spectrometry raw data were performed using Firmiana proteomics workstation, DIA-NN (v1.7.10), and Skyline-daily (4.2.1.19004, University of Washington, USA); Protein quantification was performed using the MaxLFQ algorithm and intensity-based absolute quantification (iBAQ) algorithm; Statistical analyses were realized by R (v3.5.1). Approaches or algorithms used for the proteome data annotation include ConsensusClusterPlus (version 3.8), clusterProfiler R package (v3.18.1), GSVA R package (v1.34.0), pROC R package version 1.16.2, Caret R package version 6.0–86, STRING version 11.5, Survminer R package (version 0.2.4). Gene annotation was performed using online tool ConsensusPathDB. Standard statistical tests were used to analyze the clinical data, including but not limited to Student's t test, Wilcoxon rank-sum test, Fisher's exact test, Pearson's correlation test, Spearman correlation test, log-rank test, one-way ANOVA. Unless otherwise specified, all statistical tests were two-sided. To account for multiple-testing, the p values were adjusted using the Benjamini–Hochberg FDR correction. Kaplan–Meier plots with log-rank test were used to describe survival analysis. All the analyses of clinical data were performed in R (v3.5.1) and GraphPad Prism 8 software. For functional experiments, each was repeated at least three times independently, and results were expressed as mean ± SD. Statistical analysis was performed using GraphPad Prism 8 software. The p values less than 0.05, 0.01, 0.001, 0.0001 were marked with *, **, ***, ****, respectively. This study did not generate custom computer code. No special code was used in this study. The related R scripts used for statistical analyses in this study have been publicly available on GitHub repository: https://github.com/buranoyanlee/Prediction and Zenodo repository: https://zenodo.org/records/10200747.

For manuscripts utilizing custom algorithms or software that are central to the research but not yet described in published literature, software must be made available to editors and reviewers. We strongly encourage code deposition in a community repository (e.g. GitHub). See the Nature Portfolio guidelines for submitting code & software for further information.

Data

Policy information about availability of data

All manuscripts must include a data availability statement. This statement should provide the following information, where applicable:

- Accession codes, unique identifiers, or web links for publicly available datasets
- A description of any restrictions on data availability
- For clinical datasets or third party data, please ensure that the statement adheres to our policy

The raw mass spectrometry (MS) proteomics data and parallel reaction monitoring (PRM)-MS proteomics data generated in this study have been deposited in the deposited in the ProteomeXchange Consortium (dataset identifier: PXD047207) via the iProX partner repository (https://www.iprox.cn/) under the project ID IPX0005221000. The Human Protein Atlas (HPA) IHC Staining Data and the list of protein classes could be accessed at https://www.proteinatlas.org/. Molecular Signatures Database (MSigDB) could be accessed at https://www.gsea-msigdb.org/gsea/msigdb. TIMER2.0 database could be accessed at http://timer.cistrome.org/. The ConsensusPathDB (CPDB) molecular interaction data could be accessed at http://www.consensuspathdb.org/. The STRING database could be accessed at https://cn.string-db.org/. The public datasets related to ulcerative colitis (an inflammatory bowel disease) and infection disease (such as SARS-CoV-2 infection) could be obtained from Gene Expression Omnibus (GEO, https://www.ncbi.nlm.nih.gov/geo/) with the accession number GSE11223 [https://www.ncbi.nlm.nih.gov/geo/query/acc.cgi?acc=GSE11223] and GSE207015 [https://www.ncbi.nlm.nih.gov/geo/query/acc.cgi?acc=GSE207015], respectively. The remaining data are available within the Article and Supplementary Information. NCBI human Refseq protein database could be accessed at https://www.ncbi.nlm.nih.gov/ refseq/. Source data are provided with this paper.

Human research participants

Policy information about studies involving human research participants and Sex and Gender in Research.

Reporting on sex and gender

No findings related to sex or gender in this study. For the CRC patients, there are 80 males and 36 females in the discovery cohort, and 20 males and 11 females in the independent plasma validation cohort, as well as 17 males and 14 females in the independent tissue validation cohort. For the healthy controls, there are 22 males and 44 females in the plasma discovery cohort, and 12 males and 12 females in the independent plasma validation cohort. For the multi-cancer plasma independent cohort, there were 95 patients with cancers and 20 healthy controls. Among them, for the patients with cancers, there are 49 males and 46 females; for the healthy controls, there are 12 males and 8 females.

Population characteristics

Here, we included the plasma discovery cohort composed of 116 CRC patients undergoing anti-EGFR therapy with continuous multiple treatment courses and 66 healthy controls (HCs), the plasma validation cohort composed of 31 CRC patients and 24 HCs, as well as the tissue validation cohort composed of 31 CRC patients in this study. For the CRC patients, there are 80 males and 36 females with a median age of 55.5 years (ranging from 21 to 76 years) in the discovery cohort, and 20 males and 11 females with a median age of 56 years (ranging from 29 to 77 years) in the independent plasma validation cohort, as well as 17 males and 14 females with a median age of 57 years (ranging from 25 to 76 years) in the independent tissue validation cohort. For the healthy controls, there are 22 males and 44 females with a median age of 62 years (ranging from 57 to 63 years) in the discovery cohort, and 12 males and 12 females with a median age of 55 years (ranging from 25 to 68 years) in the independent plasma validation cohort. In the plasma discovery cohort, 29 patients (25%) are poorly differentiated, 54 patients (46.6%) are moderately differentiated, and 2 patients (1.72%) are well differentiated. The ECOG performance status of 111 patients (95.7%) is assessed as 1. In the plasma validation cohort, 5 patients (16.1%) are poorly differentiated, and 17 patients (54.8%) are moderately differentiated. In the tissue validation cohort, 11 patients (35.5%) are poorly differentiated, and 16 patients (51.6%) are moderately differentiated. Other clinical parameters include serum LDH level, white blood cell count, lymphocyte number, hemoglobin, and platelet count, which are summarized in Table 1 and Table S1. For the multi-cancer plasma independent cohort, there were 95 patients with cancers and 20 HCs. Among them, for the patients with cancers, there are 49 males and 46 females with a median age of 63 years (ranging from 25 to 88 years); for the healthy controls, there are 12 males and 8 females with a median age of 53.5 years (ranging from 38 to 71 years).

Recruitment

The plasma samples used in this study were obtained from patients with CRC or healthy controls, from April, 2015 to February, 2021, were reviewed in the Shanghai Cancer Center, Fudan University (Shanghai, China). The study included a total of 756 plasma samples from the discovery cohort composed of 116 CRC patients undergoing anti-EGFR therapy with continuous multiple treatment courses and 66 healthy controls (HCs), the validation cohort composed of 31 CRC patients and 24 HCs, as well as the multi-cancer cohort composed of 95 patients with cancers and 20 HCs. In the plasma discovery cohort, we collected 89 pre-treatment plasma samples and 385 post-treatment plasma samples during continuous multiple treatment courses of anti-EGFR therapy from CRC patients. In the plasma validation cohort, 31 pre-treatment plasma samples and 46 post-treatment plasma samples from CRC patients were included in the plasma validation cohort. In addition, 31 tumor tissues and 27 paired normal-adjacent tissues (NATs) of CRC patients matched with the plasma samples were also included in this study. In the multi-cancer plasma independent cohort, 115 plasma samples were collected from 95 pre-treatment patients with cancers and 20 healthy controls.

For the CRC patients, there are 80 males and 36 females with a median age of 55.5 years (ranging from 21 to 76 years) in the discovery cohort, and 20 males and 11 females with a median age of 56 years (ranging from 29 to 77 years) in the independent plasma validation cohort, as well as 17 males and 14 females with a median age of 57 years (ranging from 25 to 76 years) in the independent tissue validation cohort. The anti-EGFR therapy regimen was given at standard dosing as described in previous studies, of which patients were given 500 mg/m2 cetuximab once-every-2-weeks combined with FOLFOX/FOLFIRI/irinotecan. The inclusion criteria were as follows: (i) diagnosis of CRC reviewed by three expert pathologists; (ii) presence of at least one measurable or unmeasurable but evaluable lesion (described according to Response Evaluation Criteria in Solid Tumors [RECIST] 1.1 by CT/MRI scanning and grouped into complete response (CR), partial response (PR), stable disease (SD), or progressive disease (PD)); (iii) presence of polymerase chain reaction (PCR)-confirmed wild-type KRAS (exon 2/3/4), NRAS (exon 2/3/4), and BRAF (exon 15) genotypes in tumor tissue before the receipt of anti-EGFR therapy; (iv)

	no history of severe heart or liver disease, psychiatric disorders, hemorrhage, or perforation of the digestive tract; (v) and an Eastern Cooperative Oncology Group performance status of 0/1 at 3 days before treatment33. Here, the ORR, defined as PR plus CR, was selected for the efficacy evaluation; patients with CR and PR were defined as sensitive (S) and those with SD and PD were defined as non-sensitive (NS).<br><br>For the healthy controls, there are 22 males and 44 females with a median age of 62 years (ranging from 57 to 63 years) in the discovery cohort, and 12 males and 12 females with a median age of 55 years (ranging from 25 to 68 years) in the independent plasma validation cohort. The enrollment criteria for HC subjects were as follows: (i) the absence of benign or malignant tumors; (ii) a qualified physical examination finding no dysfunction of vital organs and (iii) normal renal function and without albuminuria.<br><br>For the multi-cancer plasma independent cohort, there were 95 patients with cancers and 20 healthy controls. Among them, for the patients with cancers, there were 49 males and 46 females with a median age of 63 years (ranging from 25 to 88 years); for the healthy controls, there were 12 males and 8 females with a median age of 53.5 years (ranging from 38 to 71 years). In the multi-cancer plasma independent cohort, we collected 115 plasma samples, including 95 plasma samples from treatment-naive patients with various cancer types (including colorectal cancer (CRC, N = 20), lung cancer (LC, N = 15), malignant lymphoma (ML, N = 10), bladder cancer (BLCA, N = 10), breast carcinoma (BRCA, N = 15), gastric cancer (GC, N = 10), esophageal cancer (EC, N = 15)), and 20 plasma samples from healthy controls (HCs, N = 20). After collection, plasma and tissue samples were stored at -80 °C.
Ethics oversight	The studies involving human participants were reviewed and approved by the Ethics Committee of Fudan University Shanghai Cancer Center (1506147). The patients/participants provided their written informed consent to participate in this study.

Note that full information on the approval of the study protocol must also be provided in the manuscript.

Field-specific reporting

Please select the one below that is the best fit for your research. If you are not sure, read the appropriate sections before making your selection.

☒ Life sciences ☐ Behavioural & social sciences ☐ Ecological, evolutionary & environmental sciences

For a reference copy of the document with all sections, see nature.com/documents/nr-reporting-summary-flat.pdf

Life sciences study design

All studies must disclose on these points even when the disclosure is negative.

Sample size	To obtain the longitudinal and deep proteomic characterization of the colorectal cancer (CRC) response to cetuximab therapy, we performed strict screening process on CRC patients based on the anti-EGFR (cetuximab) therapy. We reviewed the CRC patients receiving anti-EGFR therapy from April, 2015 to February, 2021, in the Shanghai Cancer Center, Fudan University (Shanghai, China), and examined the corresponding plasma samples during the cetuximab treatment. The anti-EGFR therapy regimen was given at standard dosing as described in previous studies, of which patients were given 500 mg/m2 cetuximab once-every-2-weeks combined with FOLFOX/FOLFIRI/irinotecan (PMID: 18665167; PMID: 29450468; PMID: 23559149). The inclusion criteria were as follows: (i) diagnosis of CRC reviewed by three expert pathologists; (ii) presence of at least one measurable or unmeasurable but evaluable lesion (described according to Response Evaluation Criteria in Solid Tumors [RECIST] 1.1 by CT/MRI scanning and grouped into complete response (CR), partial response (PR), stable disease (SD), or progressive disease (PD)); (iii) presence of polymerase chain reaction (PCR)-confirmed wild-type KRAS (exon 2/3/4), NRAS (exon 2/3/4), and BRAF (exon 15) genotypes in tumor tissue before the receipt of anti-EGFR therapy; (iv) no history of severe heart or liver disease, psychiatric disorders, hemorrhage, or perforation of the digestive tract; (v) and an Eastern Cooperative Oncology Group performance status of 0/1 at 3 days before treatment. According to NCCN guidelines, all the CRC patients receiving cetuximab therapy included in this study were left-sided RAS wild-type metastatic colorectal cancer (PMID: 33724754; PMID: 18202412; PMID: 31117039; PMID: 25115304). Finally, we assembled a cohort (the plasma discovery cohort) composed of 116 CRC patients undergoing anti-EGFR therapy with continuous multiple treatment courses, and collected 89 pre-treatment plasma samples and 385 post-treatment plasma samples during continuous multiple treatment courses of anti-EGFR therapy from CRC patients. In addition, to validate the potential biomarkers for the longitudinal response in the multi-course treatment, we reviewed the CRC patients receiving anti-EGFR therapy in the recent two years, and adopted the same inclusion criteria, then finally collected 31 pre-treatment plasma samples and 46 post-treatment plasma samples from CRC patients in the plasma validation cohort. Further, to validate the consistency of findings uncovered in the plasma and tissue samples, we reviewed all the archival formalin-fixed paraffin-embedded (FFPE) tissues from the therapy-naïve CRC patients included in this study, and finally collected 31 tumor tissues and 27 paired normal-adjacent tissues (NATs) of CRC patients matched with the plasma samples. To explore whether the four proteins were specific to CRC diagnosis rather than other cancers, we further enrolled a multi-cancer (including other six cancer types) plasma independent cohort, and collected 95 plasma samples from treatment-naive patients with various cancer types (including colorectal cancer (CRC, N = 20), lung cancer (LC, N = 15), malignant lymphoma (ML, N = 10), bladder cancer (BLCA, N = 10), breast carcinoma (BRCA, N = 15), gastric cancer (GC, N = 10), esophageal cancer (EC, N = 15)).<br><br>For the healthy controls, the enrollment criteria for HC subjects were as follows: (i) the absence of benign or malignant tumors; (ii) a qualified physical examination finding no dysfunction of vital organs and (iii) normal renal function and without albuminuria. Finally, we included 66 healthy controls in the plasma discovery cohort, 24 healthy controls in the independent plasma validation cohort, and 20 healthy controls in the multi-cancer plasma independent cohort.<br><br>All samples were included based on the strict inclusion criteria. No statistical method was used to predetermine sample size. Written informed consent was received from all patients included in this study.

Data exclusions	No data were excluded from the analyses.
Replication	All experiments were reliably reproduced and results are represented as mean ± SD as appropriate, which is indicated in figure legends. To quality control the MS performance, the mixture of all plasma samples was measured every twenty samples as the quality control standard. The quality control standard was digested and analyzed using the same method and conditions as the CRC samples.
Randomization	The CRC samples and HC samples for proteome processing were randomized, as investigators were blinded to clinical information.
Blinding	For sample processing, consensus clustering analysis, multiple logistic regression analysis, all investigators were blinded to clinical information (including age, gender, etc.).

Reporting for specific materials, systems and methods

We require information from authors about some types of materials, experimental systems and methods used in many studies. Here, indicate whether each material, system or method listed is relevant to your study. If you are not sure if a list item applies to your research, read the appropriate section before selecting a response.

Materials & experimental systems

n/a	Involved in the study
☐	☒ Antibodies
☒	☐ Eukaryotic cell lines
☒	☐ Palaeontology and archaeology
☒	☐ Animals and other organisms
☒	☐ Clinical data
☒	☐ Dual use research of concern

Methods

n/a	Involved in the study
☒	☐ ChIP-seq
☒	☐ Flow cytometry
☒	☐ MRI-based neuroimaging

Antibodies

Antibodies used	The rabbit monoclonal antibody against CD44 (1:200, Signalway Antibody, catalog No: 48911-1), and the rabbit polyclonal antibody against GZMK (1:300, Signalway Antibody, catalog No: 40985-1) were included in this study.
Validation	The rabbit monoclonal antibody against CD44 (Signalway Antibody, catalog No: 48911-1) was validated by https://www.sabbiotech.com.cn/g-151819-CD44-Rabbit-mAb-48911.html, and the rabbit polyclonal antibody against GZMK (Signalway Antibody, catalog No: 40985-1) was validated by https://www.sabbiotech.com.cn/g-14668-Granzyme-K-Polyclonal-Antibody-40985.html.
