# Supplementary material for: Longitudinal plasma proteome profiling reveals the diversity of biomarkers for diagnosis and cetuximab therapy response of colorectal cancer
Source: Nat Commun. 2024 Feb 1;15:980. doi: 10.1038/s41467-024-44911-1 (PMC10834432; doi:10.1038/s41467-024-44911-1)
Supplement: Supplementary file 4 — Description of Additional Supplementary Files [file 41467_2024_44911_MOESM4_ESM.docx]

**Description of Additional Supplementary Files**

File Names: Supplementary Data 1

Description: **Clinical characteristics of CRC patients and healthy controls included in the discovery and validation cohorts. (A)** Clinical characteristics of CRC patients included in the discovery and validation cohorts. **(B)** Clinical characteristics of healthy controls (HC) included in the discovery and validation cohorts.

File Names: Supplementary Data 2

Description: **Proteomic matrix of the plasma discovery cohort, the plasma validation cohort and the tissue validation cohort. (A)** The protein expression matrix of healthy control (HC) and CRC plasma samples from the plasma discovery cohort. **(B)** The protein expression matrix of HC and CRC plasma samples from the plasma validation cohort. **(C)** The protein expression matrix of the tissue validation cohort after match between runs. **(D)** The protein expression at least 1 unique peptide with 1% FDR of the tissue validation cohort.

File Names: Supplementary Data 3

Description: **The differential proteomic analysis between the pre-treatment CRC and healthy control plasma samples in the plasma discovery cohort, as well as CRC tissues and paired normal-adjacent tissues (NAT) samples from the therapy-naïve CRC patients in the tissue validation cohort.** **(A)** The group of pre-treatment CRC patients and healthy controls (HC) in the plasma discovery cohort. **(B)** The differentially expressed proteins (DEPs) of pre-treatment CRC and HC groups. **(C)** The pathway alterations of pre-treatment CRC and HC groups. **(D)** The group of CRC tissues and paired normal-adjacent tissues (NAT) samples from the therapy-naïve CRC patients in the tissue validation cohort. **(E)** The differentially expressed proteins (DEPs) of T and NAT groups. **(F)** The pathway alterations of T and NAT groups. **(G)** The expression of the diagnostic biomarkers in the plasma and tissue validation cohorts.

File Names: Supplementary Data 4

Description: **The potential sensitive and resistant mechanisms and biomarkers for cetuximab treatment. (A)** The group of molecular subtypes. **(B)** The significantly up-regulated expressed proteins in the proteomic subtypes of CRC Pre-treatment samples. **(C)** The pathway alterations in the proteomic subtypes of pre-treatment CRC samples enriched by these signatures of these subtypes. **(D)** The comparison of cell types between G-III subtype and other subtypes of pre-treatment CRC plasma samples. **(E)** The list of targeted peptides that unique to the signature proteins. **(F)** The signature proteins' expression by PRM assay in the plasma discovery and validation cohorts. (**G**) The signature proteins' expression in the plasma and tissue validation cohorts.

File Names: Supplementary Data 5

Description: **The analysis of the predictive models applied for the continuous multiple courses cetuximab treatment response trajectories in the longitudinal cohort. (A)** The correlation of protein expression with multiple sampling times during cetuximab therapy courses. **(B)** The differentially expressed proteins of SSG and SNSG. **(C)** The pathway alterations of SSG and SNSG. **(D)** The dynamic trajectory of the longitudinal distribution of sensitive and non-sensitive proteins with the sampling time during cetuximab treatment. **(E)** The dynamic changes of proteins included in this predictive model and clinical therapy response during cetuximab treatment. **(F)** The signature proteins' expression in the independent longitudinal plasma validation cohorts.
